# Intestinal Clear Cell Sarcoma—A Case Presentation of an Extremely Rare Tumor and Literature Review

**DOI:** 10.3390/medicina60060847

**Published:** 2024-05-22

**Authors:** Vlad Rotaru, Elena Chitoran, Madalina Nicoleta Mitroiu, Sinziana Octavia Ionescu, Ariana Neicu, Ciprian Cirimbei, Mihnea Alecu, Aisa Gelal, Andra Delia Prie, Laurentiu Simion

**Affiliations:** 1 General Surgery Department 10, “Carol Davila” University of Medicine and Pharmacy, 050474 Bucharest, Romania; 2 General Surgery and Surgical Oncology Department I, Bucharest Institute of Oncology “Prof. Dr. Al. Trestioreanu”, 022328 Bucharest, Romania; 3 Pathology Department, Bucharest Institute of Oncology “Prof. Dr. Al. Trestioreanu”, 022328 Bucharest, Romania

**Keywords:** clear cell sarcoma, rare sarcomas, soft-tissue sarcoma, intestinal clear cell sarcoma, soft-tissue melanoma, EWSR1-ATF1 fusion protein, EWSR1-CREB1, soft-tissue melanoma

## Abstract

*Background*: Clear cell sarcoma (CCS) is an extremely rare form of sarcoma representing less than 1% of all soft-tissue sarcomas. It has morphological, structural, and immunohistochemical similarities to malignant melanoma, affecting young adults and equally affecting both sexes, and is usually located in the tendinous sheaths and aponeuroses of the limbs. Gastrointestinal localization is exceptional, with less than 100 cases reported thus far. The gene fusion of activating transcription factor 1 (ATF1) and the Ewing sarcoma breakpoint region 1 (EWSR1) are pathognomonic for clear cell sarcoma, representing the key to the diagnosis. CCS is an extremely aggressive tumor, with >30% having distant or lymphatic metastasis at the time of diagnostic, and it has a high recurrence rate of over 80% in the first year after diagnosis and a high tendency for metastatic dissemination. Given the rarity of this tumor, there is no standardized treatment. Early diagnosis and radical surgery are essential in the treatment of CCS both for the primary tumor and for recurrence or metastasis. Chemo-radiotherapy has very little effect and is rarely indicated, and the role of targeted therapies is still under investigation. *Case presentation*: We present an extremely rare case of intestinal CSS in a 44-year-old Caucasian female. The patient, asymptomatic, first presented for a routine checkup and was diagnosed with mild iron-deficiency anemia. Given her family history of multiple digestive cancers, additional investigations were requested (gastroscopy, colonoscopy, tumoral markers and imaging) and the results were all within normal limits. In the subsequent period, the patient experienced mild diffuse recurrent abdominal pain, which occurred every 2–3 months. Two years later, the patient presented with symptoms of intestinal obstruction and underwent an emergency laparotomy followed by segmental enterectomy and regional lymphadenectomy for stenotic tumor of the jejunum. Histology, immunohistochemistry, and genetic testing established the diagnosis of CCS. No adjuvant therapy was indicated. Initially, no signs of recurrence or metastasis were detected, but after 30 and 46 months, respectively, from the primary treatment, the patient developed liver metastasis and pericolic peritoneal implants treated by atypical hepatic resections and right hemicolectomy. The patient remains under observation.

## 1. Introduction

Clear cell sarcoma (CCS), also called “soft-tissue melanoma”, is an extremely rare malignant tumor and was first described by F. Enzinger in 1965. It poses diagnostic challenges due to similarities in structure, morphology and immunohistochemistry very much resembling those of malignant melanoma (MM) [1,2]. Among the common phenotypic characteristics shared with malignant melanoma are the presence of melanin, as well as the expression of melanoma-associated markers such as HMB-45, microphthalmia transcription factor (MiTF), S100 protein, and Melan-A [1]. From histological and immunohistochemical points of view, CCS and MM are almost identical, the two being differentiated by fluorescence in situ hybridization (FISH) and real-time Polymerase Chain Reaction (RT-PCR). The main key in the differential diagnosis is a reciprocal chromosomal translocation t(12;22) (q13;q12) leading to the occurrence of a fusion between two genes, namely the activating transcription factor 1 (ATF1) gene and the Ewing sarcoma breakpoint region 1 (EWSR1) gene, resulting in a fusion protein, EWSR1-ATF1. The presence of this gene fusion is a relevant indicator, as it occurs in the vast majority of patients. A variant fusion protein EWSR1-CREB1 can occur in gastrointestinal CCS resulting from the chromosomal translocation t(12;22) (q34;q12). Also, MM frequently presents BRAF mutations, which are absent in CCS.

CCS originates from the neural crest cells (as proven by the presence of melanosomes in the cytoplasm of tumoral cells), and represents less than 1% of all soft-tissue sarcomas, affecting young adults and equally affecting both sexes, and is usually located in the tendinous sheaths and aponeuroses of the limbs. In addition to MM, other differential diagnoses are made with Kaposi’s sarcoma and malignant peripheral nerve sheath tumor (MPNST). Kaposi’s sarcoma typically occurs in immunocompromised patients, while MPNST is often found in patients with neurofibromatosis type 1 [3].

The most common clinical presentation is a painful (pain present in 33–55% of cases [4]), rapidly growing tumoral mass located around the ankles. More than 90% of all CCS are in the extremities and the neck. There are also rare reports of CCS located inside the thoracic and abdominal cavity. Only 6–7% of CCS cases originate from the gastro-intestinal tract, making it an extremely rare form. To the best of our ability, we could only locate fewer than 100 cases in the international literature, most being solitary tumors and presenting as intestinal occlusion or anemic syndrome. Such tumors usually present with various nonspecific digestive symptoms (such as diffuse pain, colic, nausea, or vomiting) or general signs (fatigue, weight loss). Palpable abdominal masses are also possible.

Differential diagnostics of CCS with gastro-intestinal localization include MM, malignant gastrointestinal neuroectodermal tumors or GNET (which also present EWSR1-ATF1 or EWSR1-CREB1 fusion and S100 positivity, but lack melanocytic markers and frequently have gigantic osteoclast-like cells), clear-cell carcinomas (but these are cytokeratin-positive), gastrointestinal neuroendocrine tumors (differentiated by serologic test and scintigraphy), intestinal adenocarcinomas, and gastrointestinal stromal tumors (GIST).

Given the highly heterogeneous clinical presentation, clinicians, especially those without experience, can easily be misled, struggling to differentiate clear cell sarcoma from malignant melanoma correctly, especially for the intraabdominal localization. This, combined with the fact that immunohistochemistry cannot distinguish between the two types of tumors, means that clear cell sarcoma is misdiagnosed as malignant melanoma. Due to CCS rarity, FISH and RT-PCR testing are essential for an accurate diagnosis [3]. Correct and rapid diagnostics are necessary for insuring the optimal therapeutic response and a better oncological outcome, and physicians should always consider genetic testing for CCS when faced with an MM diagnostic.

CCS is an extremely aggressive tumor, with >30% of cases having distant or lymphatic metastasis at the time of initial diagnostic and a recurrence rate of over 80% in the first year after primary treatment. More than 60% of all cases will develop metastasis within 12 months from diagnostics. Several risk factors have been described for clear cell sarcoma, in the absence of a clear cause, including chemotherapy, radiotherapy, and genetic predisposition [4,5]. In the literature, we could only find a few studies focusing on prognostic factors and survival after CCS [6,7,8]. The largest of those studies, including 489 patients, determined that 38% of patients had distant organ metastases at diagnosis (with the most common site being the lung). At diagnosis, only a third of patients were in stage I. The same study calculated a median overall survival of 57.2 months, with 5- and 10-year survival rates of 50 and 38%, respectively. Patients with localized disease had better 5- and 10 years survival rates than those with regional dissemination (82.4%, respectively, 68.8% vs. 44%, respectively, 32.5%) and none of the patients with distant dissemination survived at 5 years [8]. In addition to regional and distant dissemination, there are other prognostic factors associated with the reduction in specific disease-free survival (DFS) and overall survival (OS) for patients with CCS. A diminished DFS was associated with tumors larger than 5 cm (median DFS, 7.5 vs. 25.5 months, *p* = 0.0043), positive surgical margins (median DFS, 3.5 vs. 13 months, *p* = 0.0233), and a neutrophile–lymphocyte ratio greater than 2.73 (median DFS, 7.5 vs. 25.5, *p* = 0.0009). Similar reduced OS rates were associated with a tumor size larger than 5 cm (median OS, 23.5 vs. 63 months, *p* = 0.0075), positive surgical margins (median OS, 21.5 vs. 63 months, *p* = 0.0101), a neutrophile–lymphocyte ratio greater than 2.73 (median OS, 26 vs. 85 months, *p* = 0.0126), a lymphocyte–monocyte ratio smaller than 4.2 (median OS, 26 vs. 85 months, *p* = 0.0445) and a thrombocyte–lymphocyte greater than 103.89 (median OS, 26 vs. 85 months, *p* = 0.0147) [8].

Given the rarity of this tumor, there is not a standardized treatment. Radical surgery is essential in the treatment of CCS both for the primary tumor and for recurrence or metastasis. Although complex personalized therapies are available to all citizens based on national health insurance programs [9,10], unfortunately, for this kind of tumor personalized medicine has very little to offer except surgery. Given the rarity of this tumor, there is not a standardized treatment for CCS. Neither ESMO nor NCCN guidelines discuss CCS separately, and only offer general principles for treatment for all soft-tissue sarcomas. Radical surgery is the “gold-standard” in the treatment of CCS both for the primary tumor and for recurrences or metastasis. For a favorable prognosis, early diagnosis followed by surgical treatment with a radical approach is necessary [11]. There are no studies which evaluate the benefits of chemo- and radiotherapy in CCS patients, either in neoadjuvant or adjuvant settings. Adjuvant chemoradiotherapy may improve DFS, but without affecting OS. Systemic therapy is used for unresectable or metastatic cases and is anthracycline-based. Radiation therapy is of little use in gastrointestinal CCS.

As for targeted therapies, none are currently approved for human use and no specific therapeutic agents directly target EWSR1-CREB1 and EWSR1-ATF1 fusion proteins. However, there are a few small studies investigating IGF1R inhibitors for tumors exhibiting EWSR1 fusion proteins [12,13]. EWSR1-ATF1 fusion proteins have been reported to upregulate the expression of MET [14] and MiTF [15], a transcription factor that has been shown to drive MET expression [16,17]. This raises the possibility of using the antibody AMG102 (which suppresses MET signaling) for treating CCS. Using sunitinib for patients with CCS harboring EWSR1-ATF1 fusion has been shown to elicit a therapeutic response, but it is not clear if other genetic alterations are present [18,19]. Combination treatment with crizotinib and pazopanib can determine a durable partial response in a patient with metastatic GNET tumors harboring EWSR1-CREB1 fusion by an unknown mechanism [20]. All these represent potential directions for future research, together with potential therapies targeting STAG2 and MYC gene alterations. As can be observed in our patient NGS’s response, several other genetic alterations were identified but thus far their significance is still unknown; it could possibly represent ways of targeting SCC.

## 2. Case Presentation

A 44-year-old Caucasian female, with known hereditary predisposition to digestive neoplasms (the mother had colon neoplasm and the father had gallbladder neoplasm), asymptomatic, was diagnosed with mild iron-deficiency anemia on routine checkup in August 2018. In response, endoscopic exploration was ordered and both gastroduodenoscopy and colonoscopy results were found to be normal. Given the oncologic history in the patient’s family, an abdominal tomography scan and entero-MRI (magnetic resonance imaging) were performed, revealing a nonspecific slightly thickened jejunal parietal area without signs of upstream stasis, and no obvious tumors. Digestive tumoral markers were examined and found to be normal. As a result, the patient was scheduled for periodic follow-up.

In the subsequent period, the patient experienced mild diffuse recurrent abdominal pain that did not significantly impact daily routine or sleep quality, and which occurred every 2–3 months.

**Figure 1:**
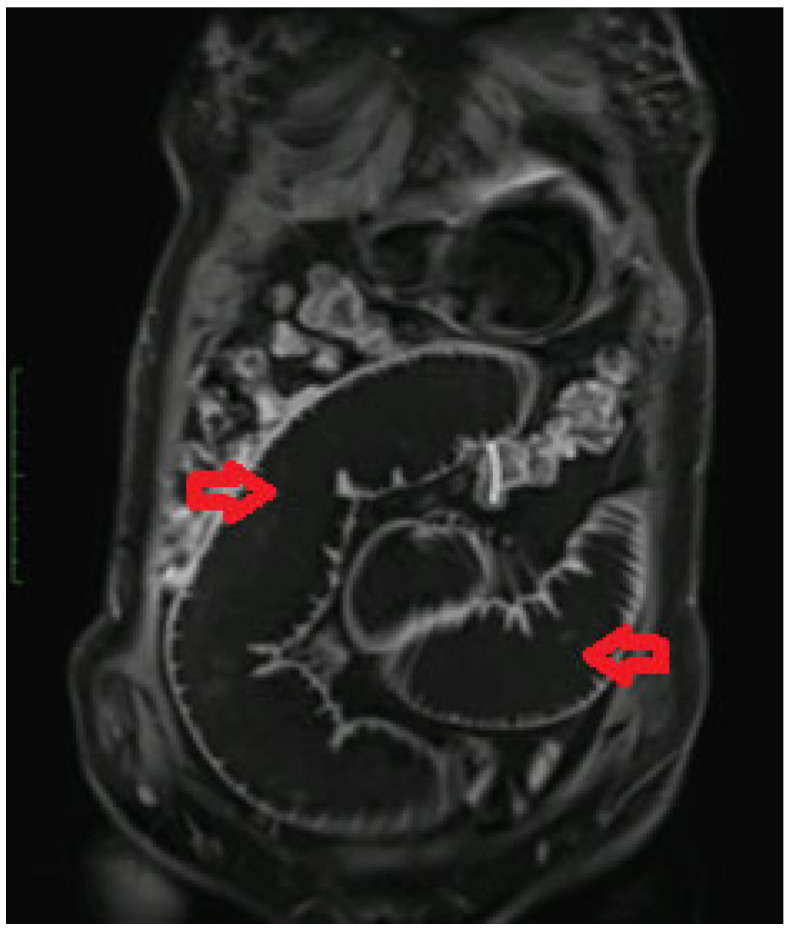
Entero-MRI which revealed a high intestinal obstruction with multiple distended jejunal loops visible marked by red arrows.

In May 2020, the patient became abruptly symptomatic, presenting with predominantly nocturnal bilious vomiting, followed by colicky pain. These symptoms recurred every 3–4 days, accompanied by repetitive episodes of belching, hiccups, a sensation of fullness, and diffuse abdominal gurgling, more pronounced in the upper abdominal region. The patient also reported a progressive weight loss of approximately 12 kg in one month in the context of voluntary limitation of food and liquid intake. The symptoms suggested an intermittent ileus and grew in intensity over time. The patient underwent multiple gastroenterology consultations, and the symptoms prompted the performance of an entero-MRI, which revealed a high intestinal obstruction due to a tight stenosis at the level of a jejunal loop (Figure 1). The presence of an invagination was maintained throughout the examination at the level of the described stenotic lesion. Small satellite lymph node images measuring 7/5 mm were also observed. Biochemical investigations revealed hypopotassemia and a slight coagulation deficiency. A complete panel of tumoral markers (consisting of CA19-9, CEA, CA125, CA15-3, CA72-4, AFP, NSE, and SCC) were requested and found to be within normal limits.

**Figure 2:**
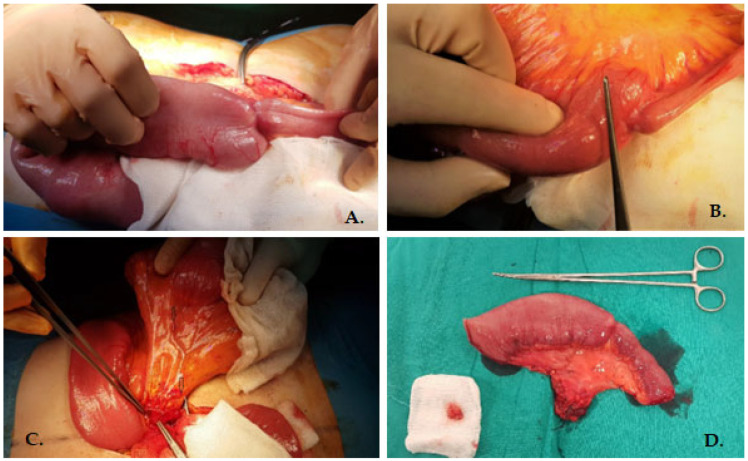
(**A**) Intraoperative image of the circumferentially thickened jejunal loop, leading to significant dilation upstream of the jejunal loops. (**B**) Dilated small intestinal loops with a diameter of approximately 5 cm. (**C**) Intraoperative aspect showing the mesentery of tumoral intestinal loop with enlarged lymph nodes (black arrows) and vascular pedicle identified at the origin from mesenteric artery, and dissected (forceps). (**D**) Resection specimen—ileal segment with corresponding mesentery.

In these conditions, with the diagnosis of intestinal obstruction, an emergency surgical intervention was performed. Upon entering the peritoneal cavity, the following were observed: dilated small intestinal loops with a diameter of approximately 5 cm, thickened jejunal walls, and liquid content up to the level of a jejunal stenosis located about 80 cm from the duodeno-jejunal angle and approximately 120 cm from the ileocecal valve. Additionally, numerous lymph nodes with a maximum diameter of 1.5–2 cm were noted along the course of the vascular bundle associated with the affected loop, without definitive macroscopic cancer characteristics (Figure 2).

Under these circumstances, a segmental enterectomy was performed with isolation of the vascular bundle supplying the affected loop at its origin and lymphatic clearance. The resection was carried out within oncological safe limits, with a 10 cm margin on each side of the stenosis of unspecified etiology. The restoration of digestive continuity was achieved through a mechanically assisted side-to-side entero-enteral anastomosis, followed by closure of the mesenteric gap.

The patient was discharged after a favorable postoperative course, with the complete resumption of intestinal transit on the 5th postoperative day, in a satisfactory general condition, afebrile, and with balanced hemodynamic and respiratory status.

The resected specimen (bowel segment and mass) was analyzed. Macroscopically, the pathologist noted a fragment of the small intestine with a dilated proximal portion and an area of vegetative tumor aspect measuring 1.5/1.5/1.2 cm in depth, covered with focally ulcerated, congested mucosa. On section, the tumor had a white color, firm consistency, and predominantly submucosal location with ulceration of the serosa. It covered approximately 30% of the lumen. Additionally, the resected triangular mesenteric portion, measuring 8/8 cm, had numerous small-sized lymph nodes.

Microscopy revealed the resection with clear proximal and distal margins. The intestinal wall showed tumor proliferation with epithelioid and spindle-shaped cells, with alveolar, solid, or storiform architecture. No necrosis was highlighted. Tumor emboli were detected in capillary-caliber vessels of the submucosa. The submitted surgical specimen contained 23 intact lymph nodes (chronic nonspecific lymphadenitis with follicular hyperplasia) and 1 lymph node with a small area of hypocellular fibrosis.

Immunohistochemistry: vimentine positive, CD99, CD15 and CD56 positive, S100 and SOX10 positive, Synaptophisin positive, negative for HMB45, MART-1, Cytokeratine, Desmin, Actin, DOG-1, CD34, CDX-2, CKIT/CD117; Ki67 35%.

**Figure 3:**
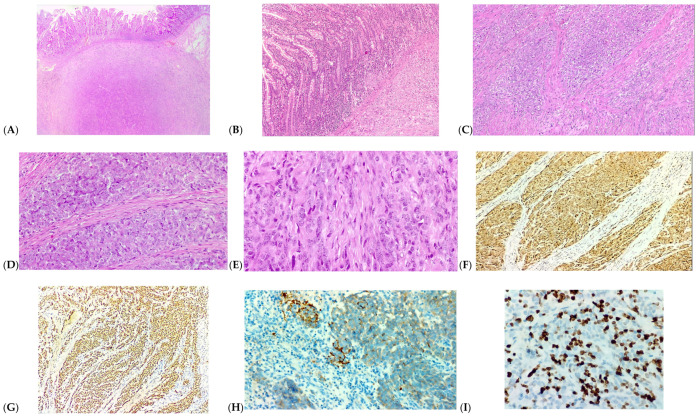
Histologic and immunohistochemical findings. (**A**) Tumor cells expanding the submucosa (HE, ×25). (**B**) Tumor cells focally infiltrating the mucosa (HE, ×100). (**C**) Atypical cells dissecting muscularis propria (HE, ×100). (**D**) Tumor cells with predominantly epithelioid morphology, prominent nucleoli, and reduced pleomorphism (HE, ×200). (**E**) Tumor cells with spindle morphology (HE, ×400). (**F**) Tumor cells are diffusely positive for S100. (**G**) Tumor cells are diffusely positive for SOX10. (**H**) Focal positivity for synaptophysin (Anti-Synaptoptophysin Ab, ×400). (**I**) High proliferation index (Ki67), about 35% (IHC, Anti-Ki67 Ab, ×400).

Histologic and immunohistochemical findings are summarized in Figure 3.

The definitive diagnosis and further therapeutic approach were determined concomitantly with the histopathological diagnosis. The histopathological results and immunohistochemical profile support the diagnosis of gastrointestinal malignant neuroectodermal tumor/clear cell sarcoma of the gastrointestinal tract (CCS).

The case was then forwarded to an oncologist for further treatment and follow-up. Given that this diagnosis is a rare one, with few cases described in the literature and limited information about therapeutic approaches for adjuvant therapy, a PET-CT was recommended to determine the actual extent of the disease, and revealed no additional tumoral sites. Additionally, a repeat of the immunohistochemistry and analyses of tissue by next generation sequencing (NGS) or Foundation One were recommended and performed. Furthermore, the patient was advised to seek consultation at other international institutes specializing in sarcomas/rare diseases for case analysis and therapeutic guidance.

Genetic testing: microsatellite stable; tumor mutational burden 4 Muts/Mb; BRAF mutations absent; NGS-based assay result showed alteration to the MYC gene, alteration of the EWSR1 gene, with EWSR1-CREB1 fusion present, and alteration to the STAG2 gene. Further, more genetic variants of unknown significance (VUS) were detected in this patient’s tumor. These variants may not have been adequately characterized in the scientific literature at the time this report was issued, and/or the genomic context of these alterations makes their significance unclear, and yet it was decided to include them in this report in the event that they become clinically meaningful in the future. BCOR-V679I; CDK12-P1257del, CREBBP-S128C; ERBB3 rearrangement, IRS2-K1170R; KDM5C-R1435C; MLL2-L4077F; SMO-Q745R.

The patient received secondary medical opinions from multiple international medical facilities specializing in sarcomas/rare diseases in Turkey, the USA, Austria, Greece, and Spain. No clinic provided an indication for adjuvant treatment; the only recommendation was regular follow-up.

The CT scans and MRIs during the first two years of follow-up did not detect any signs of tumor recurrence or metastasis. However, 30 months after primary treatment, the patient developed a unique liver metastasis which was treated by atypical hepatic resection. Similarly, at 46 months, her topography scan showed liver nodules suggestive for secondary implants and some peritoneal nodules in the vicinity of the cecum. The patient underwent laparotomy, and multiple atypical hepatic resections with right hemicolectomy were performed. The pericolic peritoneal nodules were also surgically removed. The postoperative histopathological findings showed aspects compatible with clear cell sarcoma in pericolic nodules (thus confirming peritoneal implants) and in two of the four liver nodules resected. The postoperative course of the patient was marked by a low-flow biliary fistula resulting in a small perihepatic collection which was drained under tomographic guidance. Afterwards, the evolution of the patient was uneventful.

Given the fact that the disease had progressed, systemic therapy was considered upon discussion of the case in a multidisciplinary tumor-board and will be administered to the patient. Regular follow-ups are scheduled.

## 3. Discussion

In the light of our case, we conducted an extensive search of relevant scientific publications in PubMed and Embase databases and performed a review aimed at highlighting the rarity of this type of tumor and the diagnostic and treatment challenges it raises. All peer-reviewed research and conference papers that describe a CCS with a gastrointestinal localization of tumor and genetic conformation were considered eligible for inclusion. We excluded all reports of non-digestive tumors and reported cases in which FISH or RT-PCR for EWSR1-ATF1 or EWSR1-CREB1 translocations were not performed, thus not allowing for certain diagnostics. We also excluded references that were not available in English, at least in abstract, but included references in all other languages which also had an abstract in English. We searched PubMed from inception until 15 May 2024 using relevant keywords connected by appropriate Boolean operators under the following syntax: (clear cell sarcoma) AND (gastrointestinal OR gastro-intestinal OR abdominal OR intra-abdominal). A similar search strategy was used for Embase search. Additional references were found through a rigorous citation search.

After this search, we obtained 305 results in PubMed and 136 results in Embase. An additional 23 records were identified via reference screening. All records were written between 1976 and 2024. All records retrieved were electronically screened and duplications were removed. Subsequently, two independent reviewers screened the records and excluded records that did not meet our inclusion criteria, had irrelevant focus, or presented cases already included. Discrepancies were solved via group discussion and a senior reviewer’s opinion was taken into consideration in case of disagreement. In total, 41 records were included in this systematic review. Table 1 summarizes the records included.

This study contains a qualitative but not a quantitative summary of the findings, because of the expected high heterogeneity of the articles included. Also, most studies were case reports or very small series of cases. Outcomes were reported in very few patients and, as a result, a proper statistical analysis was impossible.

The male/female ratio was 33/29. The average age was 40.84 years (with extremes of 10–85). Most cases reported small bowel tumors, followed by colonic, gastric, and pancreatic cancers. All cancers were S100 positive, but the majority had negative melanocytic markers (HMB-45, Melan-A). The clear cell sarcomas in the literature had different immunohistochemical signatures, but they all exhibited a translocation or a rearrangement of the Ewing sarcoma breakpoint region 1. In cases where data regarding the outcomes were available, we observed a high aggression of this type of tumor with a tendency to local recurrence and systemic dissemination.

## 4. Conclusions

Intra-abdominal CCS is an extremely rare tumor, most often misdiagnosed as a MM due to the clinical, histological, and immunohistochemical similarities between the two forms. Our case highlights the difficulty of the diagnosis, which requires firstly that the physician is aware of this pathology and secondly requires very specific genetic testing which is not always available in every institution (in our case, genetic testing was performed outside the country).

Also, the case presented highlights the limited therapeutic options; radical surgery remains the therapeutic “gold-standard” for both primary tumor and recurrent/metastatic disease. Our case demonstrates that a correct surgical technique, with excision with clear margins, can yield good results, as evidenced by a long-term follow-up without tumor recurrence or distant metastasis despite the lack of adjuvant therapy.

In the case of our patient, extensive genetic testing was available which, besides confirming the diagnosis, did in fact show multiple other genetic variants of unknown significance. These variants have not been adequately characterized in the scientific literature at the time of diagnosis, and/or the genomic context of these alterations makes their significance unclear but, in the future, they may become clinically meaningful or provide therapeutic options as possible targetable mutations.

## Figures and Tables

**Table 1:** Characteristics of all 62 cases already reported in the literature at the time of the study.

Record	Year	Age	Sex	Location	S-100	HMB-45	Melan-A	Other IHC Findings	Genetic Findings	Outcomes
Donner [21]	1998	37	M	Ileum	+	−	ND		EWSR1-ATF1	Liver metastasis at 24 and 36 months
Fukuda [22]	2000	74	M	Colon	+	+	ND		EWSR1-ATF1	Liver metastasis at 9 months
Pauwels [23]	2002	30	M	Stomach	+	−	−	+ for vimentin, NSE, CD99 − for cytokeratins, EMA, CD34, CD117, SMA, desmin	EWSR1-ATF1	LN and peritoneal metastasis at diagnosis; AWD at 18 months
Zambrano [24]	2003	15	F	Jejunum	+	−	−	− for CD117, CD34	EWSR1-ATF1	DOD 16 months
Achten [25]	2005	57	M	Jejunum	+	+	+	+ tyrosinase − for cytokeratins, EMA, chromogranin, CD3, CD117	EWSR1 rearrangements	NS
Venkataraman [26]	2005	21	F	Ileum	+	−	−	− for SMA, tyrosinase, CD34, CD117	EWSR1-ATF1	NS
Covinsky [27]	2005	47	F	Pancreas	+	+	+		EWSR1-ATF1	NED after 24 months
		85	F	Small intestine	+	+	+		EWSR1-ATF1	DOD at 1 month
Taminelli [28]	2005	35	M	Ileum	+	−	+	+ tyrosinase − CD117, cytokeratins, EMA, SMA, desmin, CD31, CD34, chromogranin, synaptophysin	EWSR1-ATF1	Liver metastasis at 2 months DOD 15 months
Friedrichs [29]	2005	41	M	Jejunum	+	−	−	+ vimentin, beta-catenine, CD68, PDFG-R alfa − for CD117, CD34, desmin, SMA, chromogranin, synaptophysin, NSE	EWSR1 rearrangements	Liver metastasis at 6 months
Huang [30]	2006	40	M	Stomach	+	−	−	− for CD117, CD34, vimentin, SMA, synaptophysin	EWSR1-ATF1	NS
Antonescu [31]	2006	81	F	Colon	+	−	−		EWSR1-CREB1	Liver and peritoneal metastasis at 60 months
		42	F	Ileum	+	−	−		EWSR1-CREB1	NS
		42	F	Ileum	+	−	−		EWSR1-CREB1	Liver and peritoneal metastasis at diagnosis
Granville [32]	2005	16	M	Ileum	+	−	ND	− for pancytokeratin, CD3, CD34, CD117, EMA, desmin, SMA	EWSR1-ATF1	DOD 15 months
Comin [33]	2007	31	F	Ileum	+	−	−	− for tyrosinase, cytokeratins, EMASMA, CD34, CD31, CD117, CD99, Synaptophysin, Chromogranin A	EWSR1-ATF1	NS
Abdulkader [34]	2008	37	M	Jejunum	+	+	ND	+ PDGF-R alfa, EMA, NSE, vimentine − CD34, CD117	EWSR1 rearrangement	Liver metastasis at 2 months
Lyle [35]	2008	46	M	Jejunum	+	+	+		EWSR1-ATF1	NED 7 months
		48	M	Cecum	+	+	+		EWSR1-ATF1	DOD 2 months
		60	M	Jejunum	+	+	+		EWSR1-ATF1	DOD 28 months
		62	M	Ileum	+	+	+		EWSR1-ATF1	DOD 12 months
Lagmay [36]	2009	10	F	Stomach	+	−	−		EWSR1-ATF1	NED 4 months
Joo [37]	2009	60	M	Ileum	+	−	−		EWSR1 rearrangement	NS
		46	M	Jejunum	+	−	−		EWSR1 rearrangement	NS
Terazawa [38]	2009	20	F	Ileum	+	ND	ND		EWSR1-ATF1	NED at 24 months
Shenjere [39]	2011	53	F	Ileum	+	−	−	+ for vimentin, CD57, EMA, MiTF − for CD34, DOG1, CD99, SMA	EWSR1-ATF1	Regional LN metastasis at diagnosis/NED at 7 months
		26	F	Small and large bowel	+	−	−	+ for EMA − for cytokeratins, CD99, chromogranin, synaptophysin, desmin, CD34	EWSR1-CREB1	NS
		66	M	Small intestine	+	−	−	− for cytokeratins, chromogranin, Synaptophysin, CD56, CD34, CD117, desmin, SMA	EWSR1-CREB1	Regional LN metastasis at diagnosis/NED
Balkaransingh [40]	2011	15	M	Ileum	ND	ND	ND		EWSR1 rearrangement	NS
Yang [41]	2012	15	M	Ileum	+	ND	ND	+ for vimentin	EWSR1 rearrangement	Liver metastasis at 12 months
Stockman [42]	2012	30	F	Jejunum	+	−	−	+ for SOX10, CD56, NSE, synaptophysin	EWSR1-ATF1	AWD at 21 months
		35	M	Jejunum	+	−	−	+ for SOX10, CD56, NSE, sinaptophysin	EWSR1-ATF1	DOD at 18 months
		33	M	Ileum	+	−	−	+ for SOX10, CD56 − for synaptophysin, NSE	EWSR1-CREB1	AWD at 1.5 months
		50	F	Stomach	+	−	−	+ for SOX10, synaptophysin − for CD56, NSE	EWSR1-ATF1	AWD at 24 months
		20	F	Small intestine	+	−	−	+ for SOX10, CD56, NSE − for synaptophysin	EWSR1 rearrangement	NED at 20 months
		46	M	Stomach	+	−	−	+ for SOX10, CD56 − for synaptophysin, NSE	EWSR1 rearrangement	NS
		34	F	Stomach	+	−	−	+ for SOX10, CD56 − for synaptophysin, NSE	EWSR1-ATF1	DOD at 19 months
		77	F	Colon	+	−	−	+ for SOX10, CD56, NSE, synaptophysin	EWSR1-ATF1	DOD at 106 months
		17	M	Small intestine	+	−	−	+ for SOX10, CD56 − for synaptophysin, NSE	EWSR1 rearrangement	NS
		60	M	Ileum	+	−	−	+ for SOX10, CD56, synaptophysin − for NSE	EWSR1-CREB1	AWD at 36 months
		60	F	Jejunum	+	−	−	+ for SOX10, CD56 − for synaptophysin	EWSR1-CREB1	NED at 41 months
		56	M	Stomach	+	−	−	+ for SOX10, CD56 − for synaptophysin	EWSR1-CREB1	NS
		28	F	Small intestine	+	−	−	+ for SOX10, CD56, NSE, synaptophysin	EWSR1 rearrangement	DOD at 23 months
Suárez-Vilela [43]	2012	36	F	Jejunum	+	−	−	+ for CD56, vimentin, cytokeratins, EMA − for CD117, CD99, desmin, SMA, chromogranin, synaptophysin	EWSR1-ATF1	NS
D’Amico [44]	2012	69	F	Ileum	+	−	ND	+ for CD56 − for DOG1, EMA, SMA, CD117, desmin, myogenin	EWSR1 rearrangement	Liver metastasis at 6 months
Lasithiotakis [45]	2013	49	F	Jejunum	+	−	−	+ for EMA, synaptophysin	EWSR1-ATF1	NED 20 months
Huang [46]	2014	45	F	Colon	+	−	−	− for CD117	EWSR1 rearrangement	NS
Kong [47]	2014	17	M	Stomach	+	−	−	+ for vimentin − for CD34, CD117, CD99	EWSR1 rearrangement	NED 10 months
Liu [48]		76	M	Jejunum	+	−	ND	+ for CD56 − for synaptophysin	EWSR1-ATF1	NS
Thway [49]	2014	36	M	Ileum	+	−	−	+ for EMA, CD56, NSE − for SMA, desmin, CD117, DOG1, chromogranin, synaptophysin, CD34	EWSR1-CREB1	DOD 7 months; Local recurrence + metastasis of liver, peritoneum, and regional LN at DOD
Huang [50]	2015	36	M	Pancreas	+	+	+	+ for vimentin,MiTF − for cytokeratins, EMA, desmin, SMA, CD34, CD117, CD99, synaptophysin, chromogranin, CD56, NSE	EWSR1 rearrangement	Liver metastasis at 10 months. DOD at 10 months
Yegen [51]	2015	25	F	Ileum	+	−	−	+ for vimentin, beta-catenin, CD56 − for CD34, CD117, SMA, desmin, chromogranin, synaptophysin	EWSR1 rearrangement	Liver metastasis at diagnosis and at 15 months; Ovarian and peritoneal metastasis at 47 months
Raskin [52]	2015	21	M	Small intestine	+	−	−	− for MiTF, synaptophysin, CD56	EWSR1-ATF1	LN metastasis at diagnosis
Moslim [53]	2016	57	M	Duodenum and Jejunum (2 tumors)	+	−	+	− for negative for cytokeratins, chromogranin, synaptophysin, desmin, SMA, CD34	EWSR1 rearrangement	NED 30 months and then DOD 4 months later due to rapid metastatic progression
Ardakani [54]	2016	22	M	Colon	+	−	NS	− for SMA, desmin, CD34, CD117, DOG1	EWSR1 rearrangement	NS
Su [55]	2017	51	M	Ileum and Jejunum (3 tumors)	+	+	+	+ for vimentin, CD56 − for Synaptophysin, cytokeratins, CD34, CD117, DOG1	EWSR1 rearrangement	NS
Kato [56]	2017	47	F	Colon	+	−	−	+ for vimentin, SOX10 − for SMA, CD117, cytokeratin	EWSR1-CREB1	NS
Aksan [57]	2019	28	M	Small intestine	+	−	NS	+for SOX10 − for CD117, DOG1, desmin	EWSR1-ATF1	Liver and LN metastasis at diagnosis
Okada [58]	2020	38	F	Small intestine	+	−	−	+ for CD56, synaptophysin − for desmin, chromogranin, CD34, CD117, SMA	EWSR1 rearrangement	LN metastasis at diagnosis Liver metastasis at 36 months (surgery); NED at 72 months
Zhu [59]	2021	65	M	Ileum	+	+	−	+ for SOX10, MiTF − for cytokeratins, EMA, CD117, DOG1, CD34, SMA, desmin, synaptophysin, chromogranin	EWSR1-ATF1	NED at 7 months
Huang [60]	2022	16	M	Ileum	+	−	−	+ for CD34 − for cytokeratins, CD117, DOG1, desmin, NSE	EWSR1-ATF1	DOD at 56 months Liver, lung, bone, LN, pleural and adrenal metastasis at DOD
Njima [61]	2024	20	F	Ileum	+	−	−	+ for SOX10, synaptophysin − for CD117, DOG1, cytokeratins, CD34, SMA, desmin, chromogranin	ND *	NS

Abbreviations: M—male; F—female; S-100—calcium-binding protein; HMB-45—Human Melanoma Black 45 antibody; Melan-A—Melanocyte Antigen; ND—not done; NS—not specified; ND *—not done yet; DOD—date of death; NED—no evidence of disease; AWD—alive with disease; LN—lymph nodes; IHC—immunohistochemistry; NSE—neuron specific enolase; EMA—epithelial membrane antigen; SMA—smooth-muscle actin; CD—cluster of differentiation; PDGF-R—platelet-derived growth factor receptor; SOX10—Sry-related HMg-Box gene 10; DOG1—“Discovered on GIST 1” gene; MiTF—microphthalmia transcription factor; EWSR1—Ewing sarcoma breakpoint region 1; EWSR1-ATF1—translocation between Ewing sarcoma breakpoint region 1 and activating transcription factor 1; EWSR1-CREB1—translocation between Ewing sarcoma breakpoint region 1 and cAMP responsive element binding protein 1.

## Data Availability

No new data were created or analyzed in this study. Data sharing is not applicable to this article.

## Abbreviations

CCS	Clear cell sarcoma
ATF1	Activating transcription factor 1
EWSR1	Ewing sarcoma breakpoint region 1
CREB1	cAMP responsive element binding protein 1
MM	Malignant melanoma
HMB-45	Human melanoma black 45 antibody
MiTF	Microphthalmia transcription factor
S100	Calcium binding protein
Melan-A	Melanocyte antigen
FISH	Fluorescence in situ hybridization
RT-PCR	Real-time polymerase chain reaction
BRAF	v-raf murine sarcoma viral oncogene homolog B1
MPNST	Malignant peripheral nerve sheath tumor
GNET	Gastrointestinal neuroectodermal tumors
CT	Computer tomography
MRI	Magnetic resonance imaging
CA19-9	Carbohydrate antigen 19-9
CEA	Carcinoembryonic antigen
CA125	Carbohydrate antigen 125
CA15-3	Carbohydrate antigen 15-3
CA72-4	Carbohydrate antigen 72-4
AFP	Alpha fetoprotein
NSE	Neuron-specific enolase
SCC	Subfraction of tumor-associated antigens related to squamous cell carcinoma
CD99	Cluster of differentiation 99
CD15	Cluster of differentiation 15
CD56	Cluster of differentiation 56
SOX100	Sry-related HMg-Box gene 10
MART-1	Melanocyte antigen (also called Melan-A)
DOG-1 gene	“Discovered on GIST 1” gene
CD34	Cluster of differentiation 34
CDX-2	Caudal-type homeobox 2
CKIT/CD117	Receptor for tyrosine kinase
Ki67	Proliferation index
HE	Hematoxylin and eosin stain
Ab	Antibody
PET-CT	Positron emission tomography
NGS	Next-generation sequencing
VUS	Genetic variants of unknown significance
MYC gene	Myelocytomatosis oncogene
STAG2 gene	Stromal antigen 2 gene
DFS	Disease-free survival
OS	Overall survival
ESMO	European Society of Medical Oncology
NCCN	National Comprehensive Cancer Network
MET	Mesenchymal epithelial transition—tyrosine kinase receptor
